# Analysis of potential immunoglobulin E epitopes of Gly m 4, a bet V 1-related allergen in soy beans

**DOI:** 10.1186/2045-7022-1-S1-O10

**Published:** 2011-08-12

**Authors:** Dirk Schiller, Stefan Vieths, Thomas Holzhauser

**Affiliations:** 1 Paul-Ehrlich-Institut, Division of Allergology, Langen, Germany

## 

Binding and crosslinking of FcåRI-bound IgE to conformational epitopes on allergens causes hypersensitivity reactions in allergic patients. The knowledge and modulation of epitopes on the molecular level allows the design of both hypoallergenic recombinant variants of the allergen and specific antibodies for therapeutic and diagnostic purposes, respectively.

We sought to identify and analyze the IgE-binding epitopes on Gly m 4, a soy bean allergen that is also responsible for anaphylactic reactions in individuals with birch pollen pollinosis.1

Utilizing a phage-display peptide library Mittag et al. identified peptides that bound Gly m 4-specific IgE.2 In the present study we reassessed these data and mapped 21 mimotopes onto the molecular surface of Gly m 4 (PDB code 2K7H). Using an algorithm to predict conformational epitopes on the protein surface we identified 8 major patches that might represent IgE epitopes.3 To verify the in-silico predicted IgE-binding surface areas of Gly m 4 we chose a mutagenesis approach and substituted alanine for lysine residues within the putative epitopes. The resulting recombinant Gly m 4 variants were expressed in Escherichia coli and IgE-binding was tested in western blot analyses and ELISA with a panel of sera of soy-allergic individuals sensitized to Gly m 4. rGly m 4 variants with low or no IgE-reactivity were purified and their molecular integrity was analyzed by static and dynamic light scattering as well as circular dichroism spectroscopy. Studies on the capability of the rGly m 4 variants to release mediators in humanized rat basophil cells as well as a comprehensive epitope analysis of Gly m 4 screening different phage-display peptide libraries are in progress. The benefit of a thorough IgE epitope analysis of Gly m 4 for diagnosis and therapy of soy allergy is discussed.

